# Topographic relationship between glial cells and neovessels of the epiretinal membrane in proliferative diabetic retinopathy depends on the phase of angiogenesis

**DOI:** 10.3389/fncel.2025.1571596

**Published:** 2025-04-23

**Authors:** Svetlana V. Sdobnikova, Sergey S. Makhotin, Alexander V. Revishchin, Veronika Y. Sysoeva, Galina V. Pavlova, Lyubov E. Sdobnikova

**Affiliations:** ^1^Department of Aging-associated Diseases, Medical Scientific and Educational Institute of Lomonosov Moscow State University, Moscow, Russia; ^2^Department of Ophthalmology, Medical Scientific and Educational Institute of Lomonosov Moscow State University, Moscow, Russia; ^3^Laboratory of Neurogenetics and Genetics of Development, Institute of Higher Nervous Activity and Neurophysiology, Moscow, Russia; ^4^Laboratory of Tissue Morphogenesis and Repair, Medical Scientific and Educational Institute of Lomonosov Moscow State University, Moscow, Russia

**Keywords:** proliferative diabetic retinopathy, angiogenesis, glia, neovessels, epiretinal membranes

## Abstract

**Objectives:**

To investigate the topographic relationship between glial tissue and active neovessels in epiretinal membranes (ERMs) in proliferative diabetic retinopathy (PDR).

**Materials and methods:**

Phase-contrast and immunofluorescence microscopy were performed on 17 surgically removed ERMs from 17 eyes of 17 PDR patients. Clusters of active neovessels and the surrounding posterior hyaloid membrane were excised en bloc. ERMs were immunolabeled with anti-glial fibrillary acidic protein (GFAP) antibodies to identify glia, and with anti-collagen IV or anti–von Willebrand factor (VWF) antibodies to identify neovessels. All ERMs were analyzed as whole-mounted preparations, each including the area of leading neovessels.

**Results:**

GFAP-immunopositive glial cells (GCs) were identified in 11 of 17 specimens (65%). These cells also co-expressed type IV collagen. Fibrils immunopositive for type IV collagen (GFAP-negative) were detected in all cases. The topography, structure, and GFAP immunoreactivity distinguished GCs from GFAP-negative hyalocytes. GCs had bipolar shape, small cell bodies, very long, sparsely branching, bidirectional processes, and showed a tendency to form clumps. The structure of GCs was more consistent with that of Müller cells. In all ERMs, the majority of GCs were localized around the epicenter of neovascular clusters (where neovessels branched from the maternal vessel), which also corresponded to the highest density of collagen fibrils. In four cases (23.5%), GCs were also identified in the area of the leading capillaries; however, no signs of direct interaction between GCs and developing neovessels was observed in these cases.

**Conclusion:**

Our study found no evidence of direct interaction between GCs and leading neovessels in PDR, opposite to what was shown in embryonic retinal angiogenesis. The findings may suggest that the presence of GCs near the neovascular cluster epicenter and around leading capillaries reflects different phases of the proliferative process in PDR. In the first case, GFAP+ cells appear to be involved in the involution of neovessels, which occurs during vascular remodeling or regression. In the second case, when GCs were located around the leading neovessels, their proliferation was not directly related to blood vessel formation; in our opinion, these processes may represent independent events that might have common triggers.

## 1 Introduction

Treatment of diseases accompanied by the formation of epiretinal membranes (ERMs) remains one of the main challenges in modern ophthalmology. Among these conditions, proliferative diabetic retinopathy (PDR) poses the most serious threat to vision (Stitt et al., 2016). The incidence of PDR in diabetes varies from 2% to 37% (Nwanyanwu et al., 2013), depending on the type of diabetes, age of onset, duration and severity.

Pathologic angiogenesis is the main manifestation of PDR. Neovascularization is the key feature that fundamentally differentiates PDR from other common retinal diseases in adults that are also accompanied by ERM formation, such as proliferative vitreoretinopathy, epimacular fibrosis, and idiopathic macular hole.

To ensure clarity in presenting the results of this study, we have deemed it appropriate to discuss some of the terms used in this article.

1. *Neovascular cluster*. New blood vessels have a characteristic shape (Danis and Davis, 2008). Various authors used their own terms, each of which aptly described the picture observed during ophthalmoscopy of neovessels: “new vessel patch” or «new vessel complex» (Danis and Davis, 2008), “fan of new vessels” (Bek et al., 2010; Joussen et al., 2007), and “neovascular tuft” (Soliman and Larsen, 2007). Evidently, some neovessels are fan-shaped, others look like a tuft, or even a wheel-shaped net. Given these variations, in our study we opted to use a broader term — neovascular cluster — to describe a discrete set of neovessels that branch from a single location (from the retinal vessels or the optic disk).

2. Posterior hyaloid membrane and vitreoschisis. Neovessels grow along the posterior vitreous surface (Davis, 1965; Snead et al., 2002), which becomes evident when partial posterior vitreous detachment occurs. On the outside of the vitreous there is a dense layer, the vitreous cortex (Sebag, 1992; Tozer et al., 2014), surrounded by the hyaloid membrane (Snead et al., 2002; Worst, 1978). The posterior hyaloid membrane is not detectable in the eyes without vitreous detachment. Therefore, many researchers use the names such as “cortical vitreous” (Kishi and Shimizu, 1993), or “posterior hyaloid” (Muqit and Stanga, 2014) to define the preretinal layers of the vitreous body. However, once posterior vitreous detachment occurs, a distinct membranous structure becomes visible in clinical and histopathological examinations (Davis, 1965; Kishi and Shimizu, 1993; Lumbroso and Rispoli, 2012; Schwatz et al., 1996).

Removal of the posterior hyaloid membrane, along with neovessels and proliferative tissue, is a key step in vitreoretinal surgery for PDR. It is also important to note that the term “epiretinal membrane” is commonly used in various retinal diseases, including PDR. Therefore, in this study we decided to use the term “posterior hyaloid membrane.” Gärtner (1963) demonstrated in his work that the posterior hyaloid membrane consists of two layers. Worst (1978) observed that posterior vitreous detachment results in “splitting in the lamellae of the posterior vitreous membrane.” Multiple membranous structures had been visualized in numerous clinical and histopathological studies (Chu et al., 1991; Gupta et al., 2011; Kakehashi et al., 1993; Morishita et al., 2021; Schwatz et al., 1996; Sebag, 2008) following posterior vitreous detachment. This condition is known as vitreoschisis. In PDR, neovessels and proliferative tissue may spread along one or more layers of the vitreoschisis (Schwatz et al., 1996; Worst, 1978).

**FIGURE 1:**
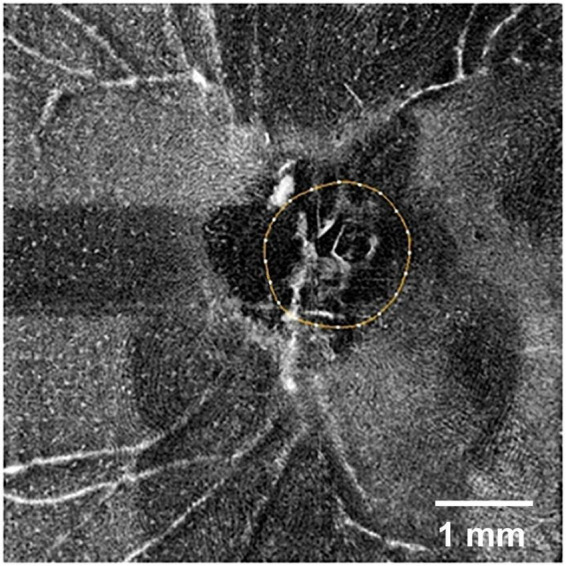
Optical coherence tomography angiography (OCTA) image of the retinal surface of a normal human eye (SOLIX, Optovue, United States; AngioVue disk mode, 6 mm). The optic disk is outlined by a circle. White dots in the image represent individual hyalocytes evenly distributed above the retinal surface. Scale bar: 1 mm.

3. Hyalocytes. Another important feature of the preretinal vitreous is the presence of hyalocytes, which are the tissue-resident macrophages of the vitreous body. These cells are localized in single layer of the vitreous cortex (Sebag, 1998), have a characteristic phenotype and relatively uniform distribution (Jones et al., 2022; Qiao et al., 2005; Sakamoto and Ishibashi, 2011). Hyalocytes can be visualized in healthy eyes using clinical examination methods (Wieghofer et al., 2022; Figure 1).

It has been demonstrated that retinal vessels and glial cells (GCs) interact during normal embryonic and perinatal angiogenesis (Chan-Ling et al., 2004; Fruttiger, 2002; Provis, 2001; Stone and Dreher, 1987). In the primary vascular plexus of the mammalian retina, capillaries replicate the pattern of the existing astrocyte network. Moreover, the filopodia of leading endothelial cells are localized along the bodies and processes of GCs (Dorrell et al., 2002). Several studies have shown that GC proliferation plays a major role in ERM formation in various human retinal diseases, including PDR (Bringmann and Wiedemann, 2009; Romaniuk et al., 2013). However, other studies suggest that GFAP-positive cells are more commonly associated with retinal diseases that do not involve neovessel formation (Bellhorn et al., 1975; Ozóg et al., 2023; Snead et al., 2008; Sramek et al., 1989; Tsotridou et al., 2020), such as proliferative vitreoretinopathy, epimacular fibrosis, and macular holes. Additionally, some authors suggest that hyalocytes, rather than GCs, play a critical role in ERM pathology (Boneva et al., 2021; Kohno et al., 2009; Sakamoto and Ishibashi, 2011; Schaefer et al., 1996; Sebag, 1989). Thus, the role of GCs in ERM formation in PDR remains a subject of debate.

The main goal of this study was to investigate the topographical relationship between glial cells and active neovessels in epiretinal membranes (ERMs) in PDR. Specifically, we examined the frequency of glial cell occurrence in ERMs, the topography of glial tissue in various zones of neovascular clusters, and the different variants of relationships between glial cells and leading neovessels.

## 2 Materials and methods

### 2.1 Patient information and acquisition of ERM specimens

**FIGURE 2:**
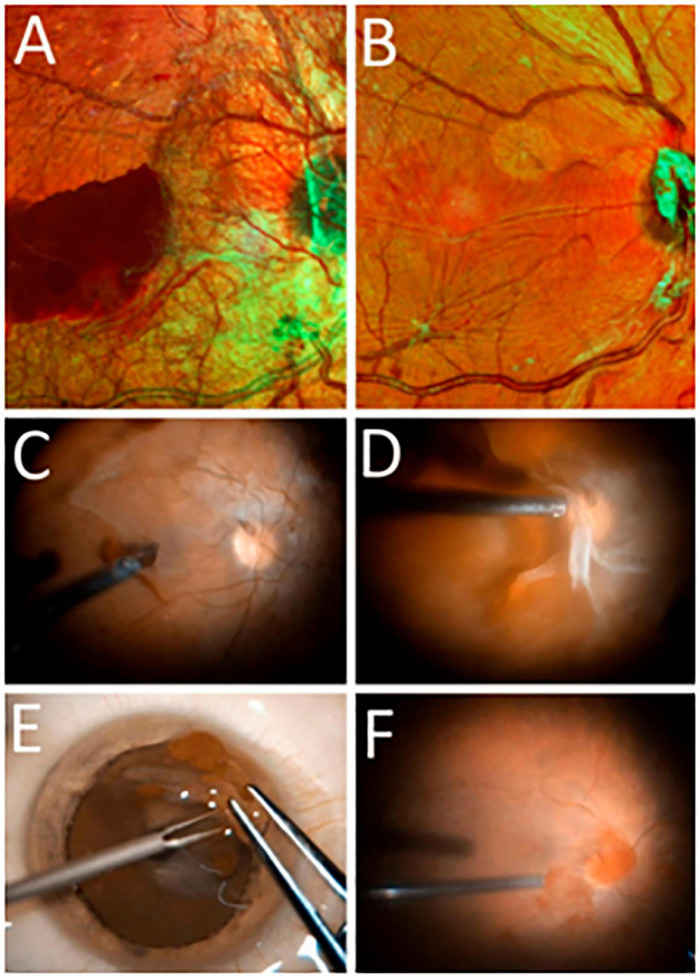
**(A,B)** Scanning laser ophthalmoscopy (SLO) images of the ocular fundus in PDR (Spectralis HRA, Heidelberg Engineering, Germany; multicolor mode): **(A)** before vitrectomy (before the ERM containing neovessels was removed) and **(B)** after vitrectomy (following ERM removal). **(C–F)** Still frames from intraoperative video: **(C,D)** separation of the posterior hyaloid membrane with ingrowing neovessels; **(E)** appearance of the excised epiretinal membrane spread out on the corneal surface; **(F)** intraoperative image of the retinal surface after epiretinal membrane (ERM) removal.

The study included 17 ERMs with active neovessels obtained from 17 eyes of 17 PDR patients during vitreoretinal surgery (Figures 2A–F); all ERMs contained the area of leading neovessels. Nine patients had type 1 diabetes mellitus (DM1) (53%), and eight had type 2 (DM2) (47%). The average age of the patients was 42 [32; 56] years. There was no statistically significant difference in sex distribution between the DM1 and DM2 groups (*P* = 0.637, Fisher’s exact test). All patients underwent standard 23-gauge three-port vitrectomy (Stellaris, Bausch and Lomb, Rochester, NY, United States) performed by a single surgeon. When possible, neovascular clusters and surrounding posterior hyaloid membranes were excised en bloc. The excised tissues were fixed in 4% paraformaldehyde for 60 min and stored in antifreeze (a mixture of glycerol, ethylene glycol, and phosphate buffer, 1:1:1) at -20°C. Patients underwent standard preoperative and postoperative examinations, including scanning laser ophthalmoscopy and fluorescein angiography when feasible (Spectralis HRA-OCT, Heidelberg Engineering, Germany). The study was conducted at the Medical Scientific and Educational Institute of Lomonosov Moscow State University from 2021 to 2024. Written informed consent was obtained from all patients. The study adhered to the principles of the Declaration of Helsinki. The study was approved by the Ethics Committee of the Medical Scientific and Educational Institute of Lomonosov Moscow State University (dated 2021-02-01).

### 2.2 Immunohistochemical staining

Epiretinal membrane specimens were analyzed using phase-contrast and immunofluorescence microscopy (Olympus IX81, Germany; Olympus DP72 digital camera, Germany). The ERMs were processed as whole mounts without embedding or sectioning. Mouse monoclonal antibodies against GFAP (Sigma-Aldrich, United States) were used to identify glial cells in all 17 specimens. To identify neovessels, rabbit polyclonal antibodies against collagen IV (Abcam, United States) were used in seven specimens to stain vascular basement membranes, and rabbit polyclonal antibodies against von Willebrand factor (VWF) (Abcam, United States) were used in 10 specimens to identify endothelial cells. Primary antibodies were diluted according to the manufacturer’s instructions, and specimens were incubated overnight at 4°C on a rotator. After nuclear staining with bisbenzimide (1.0 g/mL Hoechst 33342), specimens were washed and incubated with secondary antibodies conjugated with fluorescent dyes Cy2 or Texas Red (Jackson ImmunoResearch, United States). The ERMs were flattened onto slides using two diamond scrapers, clarified with glycerin, and covered with coverslips. Hyalocytes were identified by their characteristic: location and macrophage-like morphology using phase-contrast microscopy and nuclear counterstain (Hoechst 33342). Human retinal sections served as positive controls. In normal human retinas, immunolabelling of all used antibodies was observed. For negative control, rabbit and mouse non-immune serums were used instead of primary antibodies. No significant immunoreactivity was observed in any of the negative control samples.

### 2.3 Statistical analysis

Data were analyzed using IBM SPSS Statistics v.26 (IBM Corporation). The Kolmogorov-Smirnov test was used to assess the normality of distribution. The median, as well as the first and third quartiles were calculated for the analyzed parameters that had a non-normal distribution and measured on an interval (quantitative) scale. Fisher’s exact test was used to compare nominal variables when low frequencies were present. Spearman’s correlation coefficient was used to determine the relationship between variables.

## 3 Results

### 3.1 Terminology

Neovascular clusters with leading blood-perfused capillaries (ophthalmoscopically visible) were considered active and designated with the letter “V” (from the Latin “vitalis,” meaning living). The absence of blood-perfused neovessels indicated their involution (inactivity) and was designated with the letter “M” (from the Latin “moriens,” meaning dying); these cases were not included in the study. Because neovessel caliber varied, all cases were categorized according to the stage of angiogenesis: V1 (capillaries only), V2 (capillaries + medium-caliber neovessels), and V3 (advanced neovascular network, including large new vessels).

**FIGURE 3:**
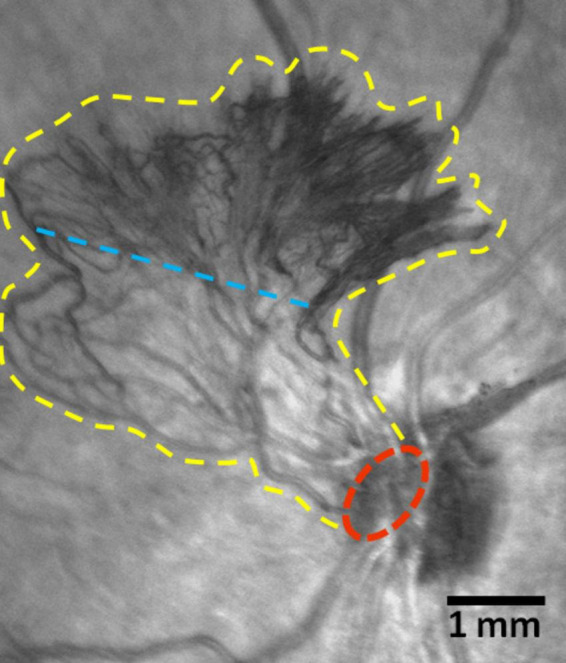
Scanning laser ophthalmoscopy (SLO) images of a fragment of the ocular fundus in proliferative diabetic retinopathy (PDR) (Spectralis HRA, Heidelberg Engineering, Germany; infrared mode). New vessels of the optic disk. The yellow line marks the boundaries of the neovascular cluster, the red line indicates its epicenter. The blue line arbitrarily separates the cluster base (below the line) comprised of medium-caliber vessels, from the growth zone (above the line), which contains leading capillary loops. Scale bar: 1 mm.

“Neovascular cluster” refers to a discrete set of neovessels branching from a single location. The “neovascular cluster epicenter” or “epicenter of neovascularization” is the point where the cluster’s neovessels branch from the maternal retinal vessel or optic disk vessel (Figure 3). The “cluster base” is the area surrounding the epicenter of neovascularization (proximal part of the cluster). The “growth zone” is the part of the neovascular cluster containing the leading loops of new vessels (distal part of the cluster) (Figure 3). “Neovascular network” is a system of clusters that form anastomoses as their area increases.

### 3.2 General characteristics of the studied ERMs

**FIGURE 4:**
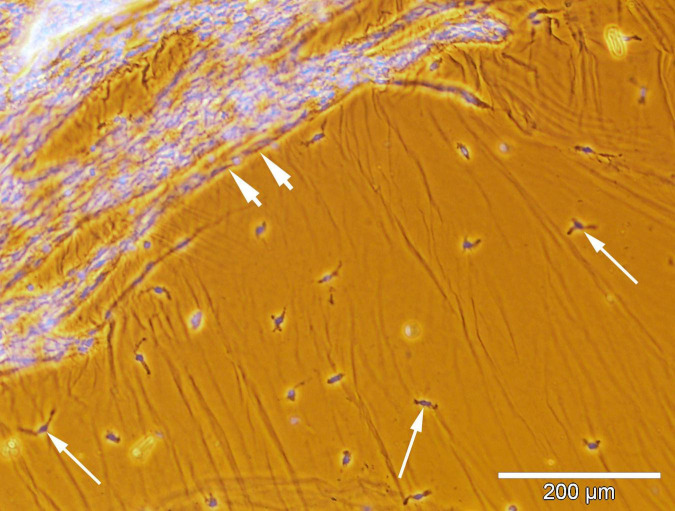
Spatial relationship between the hyalocytes and new vessels in the excised epiretinal membrane (ERM). Whole-mounted ERM specimen removed from a proliferative diabetic retinopathy (PDR) patient. Merged phase-contrast and fluorescence microscopy. Cell nuclei were stained with Hoechst 33342 (blue fluorescence). Neovessels are indicated by the arrowheads. Hyalocytes (indicated by arrows) outside the outer boundary of neovascularization are identified by their typical localization in the posterior hyaloid membrane and macrophage-like shape. Scale bar: 200 μm.

The ERMs of 17 eyes from 17 patients who underwent vitrectomy for active PDR were analyzed (Figures 2A–F). The ERMs were examined as flat whole-mount preparations. The specimens obtained during the active stage of angiogenesis revealed vascular pattern and cell topography using conventional (non-confocal) fluorescence microscopy (Figure 4).

Each specimen contained both the base of the neovascular cluster (“cluster base”) and the area of leading capillary loops (“growth zone”).

The frequency distribution of angiogenesis stages (V1, V2, V3) did not differ significantly between patients with DM1 and DM2 (*P* < 0.086, Fisher’s exact test), likely due to the small sample size and low frequency values. However, patients with DM1 predominantly exhibited later stages of angiogenesis (89% of cases), with 56% classified as V2 and 33% as V3. In DM2 patients, in 63% of cases the neovessels were at the capillary stage (V1). The frequency of late-stage neovascularization (V2 + V3) was significantly higher in DM1 than in DM2 (Fisher’s exact test, one-sided *P* = 0.027, two-sided *P* = 0.05). The distribution of ERMs by neovascularization stage and diabetes type is presented in Table 1.

**TABLE 1:** Distribution of the studied epiretinal membrane (ERM) specimens according to the stage of neovascularization and type of diabetes mellitus in patients.

Stage of angiogenesis		DM1	DM2	Total
	*N* = 9	***N* = 8**		
V1 – Capillaries	*N*	1	5	6
	%	11%	63%	35%
V2 – Capillaries and medium-caliber neovessels	*N*	5	2	7
	%	56%	25%	41%
V3 – Advanced neovascular network with large neovessels	*N*	3	1	4
	%	33%	12%	24%
Total	*N*	9	8	17
	%	100%	100%	100%

V1, V2, V3—stages of angiogenesis in the epiretinal membrane: V1, capillaries only; V2, capillaries and medium-caliber neovessels; V3, advanced neovascular network including large neovessels; DM1, type 1 diabetes mellitus; DM2, type 2 diabetes mellitus.

**FIGURE 5:**
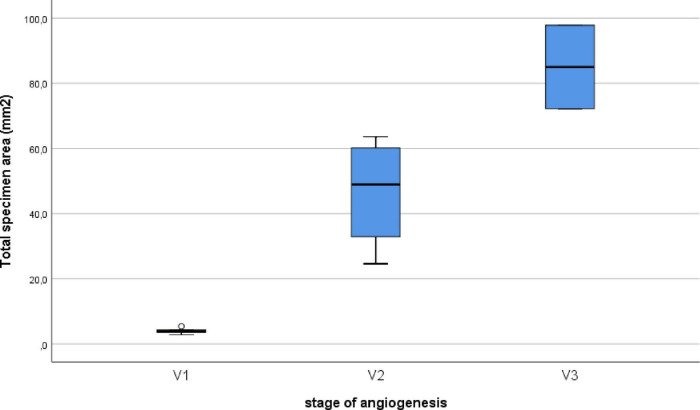
Correlation between the stage of angiogenesis and the specimen area of the ERM. A significant (*P* < 0.0005) and strong (*P* = 0.993, Spearman’s correlation coefficient) correlation was identified between the stage of angiogenesis and the area of specimen.

**FIGURE 6:**
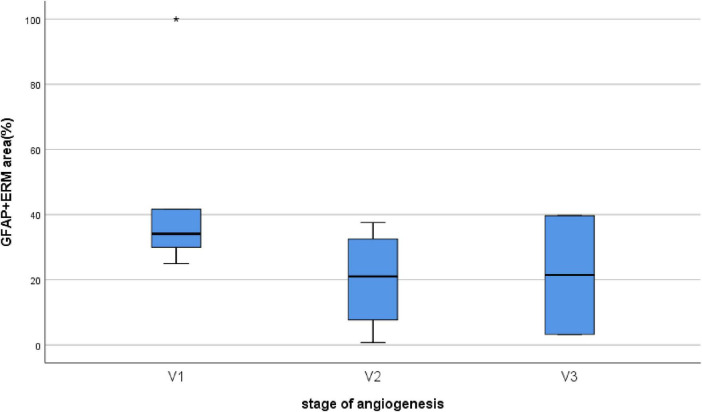
Correlation between the stage of angiogenesis and the ERM area containing GFAP+ cells. No significant correlation (*P* = 0.246) was found between the stage of angiogenesis and the ERM area containing GFAP+ cells.

Epiretinal membranes differed significantly in area depending on the developmental stage of neovessels, ranging from 2.8 mm^2^ to 97.8 mm^2^ (Table 2), with a median of 24.6 mm^2^ [3.9; 63.6]. A significant (*P* < 0.0005) and strong (*P* = 0.993, Spearman’s correlation coefficient) correlation was identified between the stage of angiogenesis and the area of specimen (Figure 5). However, no significant correlation (*P* = 0.246) was found between the stage of angiogenesis and the ERM area containing GFAP+ cells (Figure 6) or between the area of specimen and the ERM area containing GFAP+ cells (*P* = 0.385).

**TABLE 2:** Characteristics of the studied epiretinal membranes in which glial fibrillary acidic protein (GFAP)-positive cells were detected.

Pt. No.	Type of DM	Stage of angiogenesis	Antibodies	Total area of the specimen (mm^2^)	ERM area containing the GFAP+ cells (mm^2^/%)	Presence of GFAP+ cells in the cluster base	Presence of GFAP+ cells in the growth zone
1	1	V1	GFAP + ColIV	3.9	1.33/34	+	–
2	2	V1	GFAP + ColIV	3.6	1.5/42	+	+
3	2	V1	GFAP + VWF	5.4	1.62/30	+	–
4	2	V1	GFAP + ColIV	2.8	0.7/25	+	-
5	2	V1	GFAP + VWF	4.3	4.3/100	+	+
6	1	V2	GFAP + ColIV	24.6	3.6/15	+	–
7	1	V2	GFAP + VWF	56.7	21.3/38	+	+
8	1	V2	GFAP + VWF	63.6	0.48/0.7	+	–
9	2	V2	GFAP + ColIV	41.2	11.3/27	+	–
10	1	V3	GFAP + VWF	72.2	2.36/3	+	–
11	1	V3	GFAP + VWF	97.8	38.8/40	+	+

V1, V2, V3—stages of angiogenesis in the epiretinal membrane: V1, capillaries only; V2, capillaries and medium-caliber neovessels; V3, advanced neovascular network including large neovessels; DM1, type 1 diabetes mellitus; DM2, type 2 diabetes mellitus. “Neovascular cluster” - a discrete set of neovessels branching from a single location. “Cluster base” and “growth zone” denote different regions within each neovascular cluster. The “cluster base” is the area surrounding the branching point of neovessels from the maternal retinal vessel or from the optic disk vessel (proximal part of the cluster). The “growth zone” is the part of the neovascular cluster that contains the leading neovessels (distal part of the cluster).

### 3.3 Results of the ERM immunohistochemical study

**FIGURE 7:**
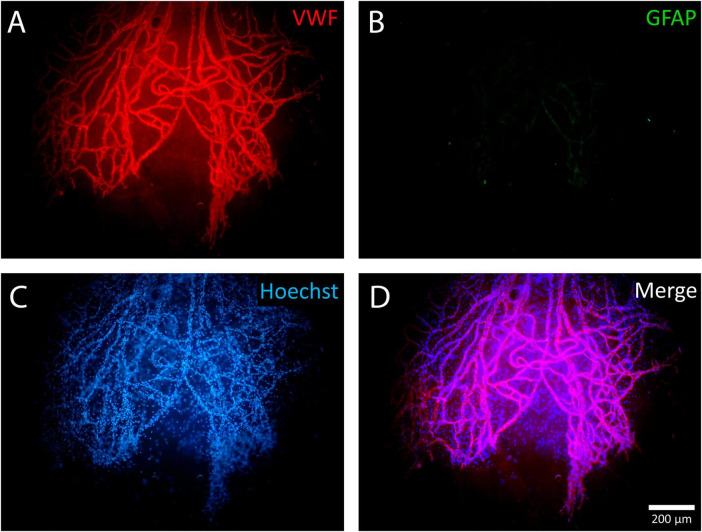
Example of a negative reaction of epiretinal membrane (ERM) cells to glial fibrillary acidic protein (GFAP). A whole-mounted ERM fragment with leading neovessels stained by immunohistochemistry: **(A)** von Willebrand factor (VWF) (red fluorescence), **(B)** GFAP (green fluorescence, negative staining result), and **(C)** Hoechst 33342 nuclear stain (blue fluorescence). When using only the nuclear stain, new vessels are clearly distinguishable due to their higher cellularity. **(D)** Merged channels. Scale bar: 200 μm.

**FIGURE 8:**
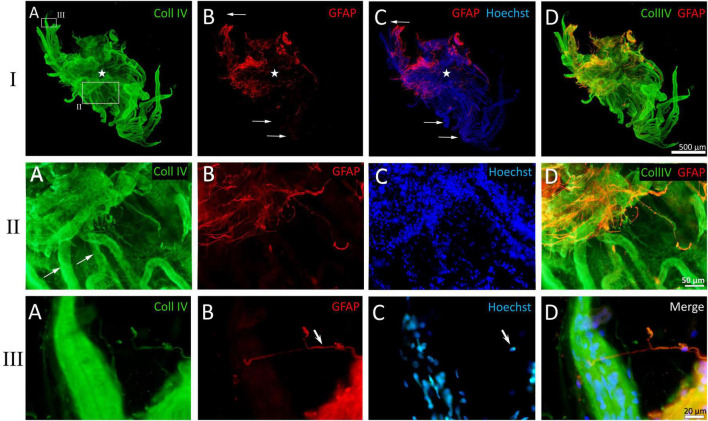
Example of a whole-mounted epiretinal membrane (ERM) specimen in proliferative diabetic retinopathy (PDR). Immunohistochemical staining for type IV collagen (green fluorescence) and glial fibrillary acidic protein (GFAP) (red fluorescence). Cell nuclei were stained with Hoechst 33342 (blue fluorescence). I. The image shows the entire neovascular cluster with the surrounding posterior hyaloid membrane. **(A)** The neovascular cluster consists of newly formed vascular loops branching from a single point (the cluster epicenter, marked by asterisks). Neovessels are well-visualized due to the immunoreactivity of their basement membrane to type IV collagen. Neovessels are surrounded by a fibrous matrix, which also contains type IV collagen-positive fibrils. The fibril density is higher at the epicenter of the neovascular cluster. The larger rectangle is shown in detail in II, while the smaller rectangle is shown in III. **(B)** Glial tissue is unevenly distributed. GFAP-positive cells are absent in the majority of distal capillary loops (arrows). **(C)** Merged channels (GFAP + Hoechst 33342). Arrows indicate the same distal capillary loops as in panel **(B)**. **(D)** Merged channels (GFAP + Coll IV). Glial cell staining demonstrates colocalization of GFAP and type IV collagen antibodies [also in II-**(D)** and III-**(D)**]. II. Detailed view of the larger rectangle from I-**(A)**. **(A)** Neovessels are marked with arrows. Type IV collagen-positive (GFAP-negative) fibrils surrounding the neovessels appear as a fine veil in the middle part of the cluster (lower part of the micrograph) and as denser tissue closer to the neovascularization epicenter (upper part of the micrograph). **(B)** GFAP-positive cells are found closer to the cluster epicenter. **(C)** Hoechst 33342 staining highlights the multicellularity of the ERM fragment. **(D)** Merged channels (Coll IV + GFAP). Colocalization of GFAP and type IV collagen antibodies is observed in glial cells. III. Detailed view of the smaller rectangle from I-**(A)**. **(A)** Distal neovascular loop. Single Coll IV-positive cell with long, bidirectional processes. **(B)** Absence of GFAP-positive tissue around the neovessel; the same glial cell (arrow) from III-**(A)** that is immunopositive for type IV collagen, is also GFAP-positive. **(C)** Cell nuclei of the vascular wall outline the contour of the neovessel. Single glial cell nucleus is visible separately (arrow). **(D)** Merged three-channel image. Scale bars: I—500 μm, II—50 μm, III—20 μm.

**FIGURE 9:**
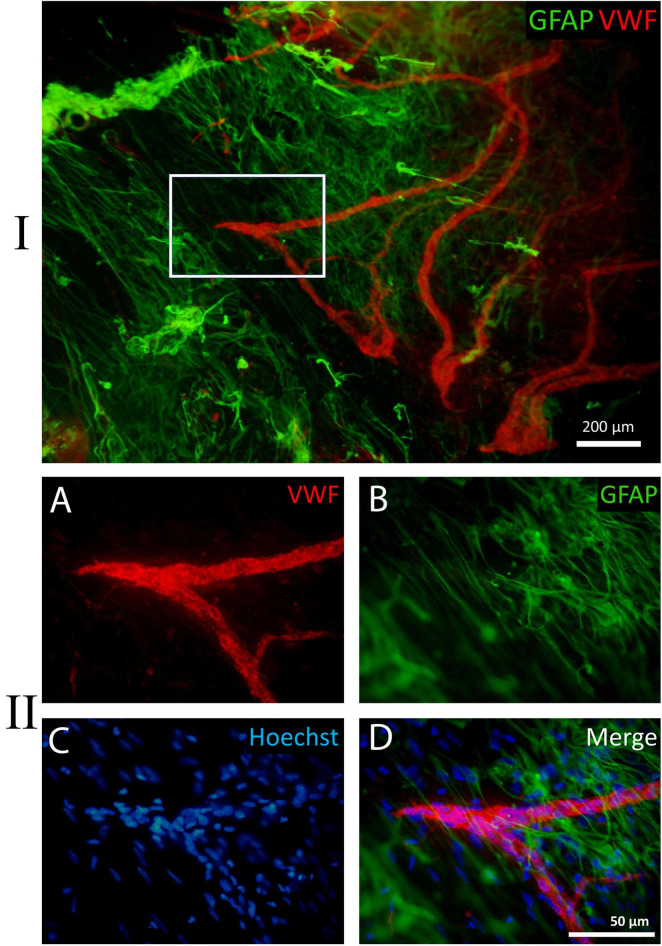
Example of a fragment of a whole-mounted epiretinal membrane (ERM) specimen in proliferative diabetic retinopathy (PDR). Immunohistochemical staining for von Willebrand factor (VWF) (red fluorescence) and glial fibrillary acidic protein (GFAP) (green fluorescence). Cell nuclei were stained with Hoechst 33342 (blue fluorescence). I. VWF-positive leading neovessels; GFAP-positive cells with long processes are located beyond the outer boundary of neovascularization. In the peripheral part of the ERM the processes of glial cells are unidirectional but do not form any clear structure. II. Detailed view of the white rectangle from I. **(A)** The direction of terminal capillary growth. **(B)** The orientation of glial cell processes, which does not align with capillary growth direction. **(C)** Cell nuclei stained with Hoechst 33342. **(D)** Merged channels. Scale bars: I—200 μm, II—50 μm.

**FIGURE 10:**
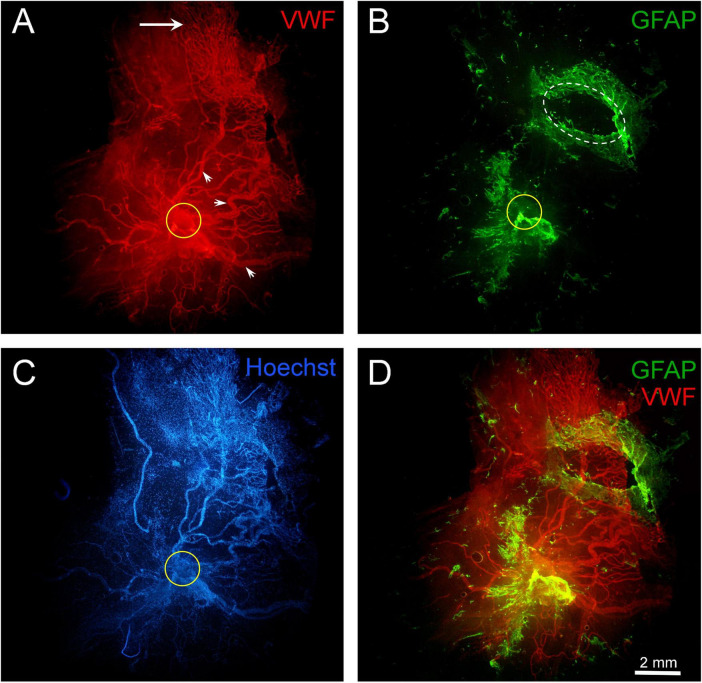
Fragment of a large epiretinal membrane (ERM) specimen (97.8 mm^2^) in proliferative diabetic retinopathy (PDR). Immunohistochemical staining for von Willebrand factor (VWF) (red fluorescence) and glial fibrillary acidic protein (GFAP) (green fluorescence). Cell nuclei were stained with Hoechst 33342 (blue fluorescence). The ERM region that was attached to the optic disk is highlighted with a yellow circle. **(A)** Immunohistochemical staining for VWF reveals the presence of very large neovessels (arrowheads) radiating from the area marked with a yellow circle (epicenter of neovascularization). The ERM contains a “mature” neovascular network, including all components characteristic of a regional hemocirculatory system: major neovessels, medium-caliber neovessels, and capillaries. The growth zone, represented by capillaries and leading neovessels (indicated by the arrow), is located in the peripheral part of the ERM. Such neovascular network may form through the merging and remodeling of individual neovascular clusters as they grow. The ERM specimen was found to separate into two layers (vitreoschisis) in the growth zone (in the upper-right quadrant of the micrograph). **(B)** GFAP-positive cells are present both at the base of the neovascular network (around the neovascularization epicenter marked with a yellow circle) and in the peripheral areas of the ERM, which contain capillaries (in the growth zone). In the upper-right part of the ERM (marked with a white oval), a fragment of one of the vitreoschisis layers was removed and examined separately; this layer contained numerous GFAP-positive cells but no neovessels. Thus, neovessels were located in one layer of the epiretinal membrane, while GFAP-positive cells were in another. **(C)** Despite the high cellularity of the ERM, nuclear staining highlights the contours of neovessels. **(D)** Channel merging (VWF + GFAP). Overlaying the channels reveals both membrane layers. The vascular network (in the lower layer) remained intact after the removal of GFAP-positive tissue fragment (from the upper layer), confirming the independence of these layers. Scale bar: 2 mm.

All 17 ERMs were multicellular, as confirmed by Hoechst nuclear staining. When only the nuclear dye (Hoechst 33342) was used, neovessels were clearly distinguishable due to their greater cellularity (Figures 7–10). Across all specimens, some cells could not be identified with the antibodies used in this study. GFAP-positive cells were absent in 6 ERMs (35%) (Figure 7).

Single, uniformly distributed macrophage-like cells were detected by phase-contrast microscopy outside the neovascular growth zone. These cells were identified as hyalocytes (Figure 4). Phenotypically similar perivascular cells were found around leading neovessels. Both cell types were GFAP-negative.

Immunostaining for type IV collagen and VWF confirmed the presence of neovessels in all ERM specimens. At advanced stages of angiogenesis, small neovessels (including leading capillary loops) were present only in the distal parts of the neovascular network, representing the growth zone. In all specimens labeled with collagen IV antibodies, the vessels were enclosed in translucent tissue containing thin, non-branching GFAP-negative collagen fibrils (Figure 8). Their density increased toward the cluster epicenter, leading to a corresponding increase in ERM thickness. In contrast, neovessels in the growth zone were more clearly visualized due to the thinner and more transparent ERM.

Glial fibrillary acidic protein-positive cells were detected in 11 of 17 specimens (65%). The area occupied by GFAP-positive cells within the ERMs varied considerably. In nine of the 11 specimens containing GFAP-positive cells, the area occupied by GFAP-positive cells ranged from 15% to 100% of the total ERM area, while in two cases, only small isolated fragments of GFAP-positive tissue were found. Table 2 summarizes the characteristics of ERMs containing GFAP-positive cells.

Across all specimens, GFAP-positive cells had a consistent phenotype: bipolar shape, small body (7–10 μm), and very long (up to 100 μm or more), sparsely branching, bidirectional processes (Figures 8, 9). Notably, GFAP-positive cells co-expressed type IV collagen (Figure 8). A characteristic feature of GCs was their aggregation into clumps, with a markedly uneven distribution in the ERM. In general, the clumps of GFAP-positive cells had a typical pattern: long GC processes of GCs were often unidirectional and were significantly thicker and less frequent compared to GFAP-negative collagen fibrils.

### 3.4 Topographic relationship between glial cells and leading capillaries in ERMs in PDR

In seven out of 11 cases, GFAP labeling was confined to the cluster base (around the branching point of the neovascular cluster from the retinal vessels) and was absent in the leading capillary loops zone. In four cases, GFAP-positive cells were detected both at the base and in the growth zone of neovascular clusters. Thus, out of all 17 specimens, GFAP-positive cells were present in the growth zone in only four cases (23.5%). In these specimens, glial tissue distribution was uneven, with a higher GC density near the cluster base. In three of the four cases, GFAP-positive cells and leading neovessels were located in different ERM layers. In two cases, GCs extended beyond the terminal capillaries.

Topography of glial cells and neovessels in the growth zone in PDR, based on those four available observations, can be summarized as follows:

Cases one and twi (specimens two and five in Table 2, respectively): ERMs with areas of 3.6 mm^2^ and 4.3 mm^2^, each containing a single angiogenesis cluster at the capillary stage (V1). Individual leading capillary loops were in contact with the processes of GFAP-positive cell. However, in adjacent fragments of the same ERM, glial cells were absent near most capillary loops (Figure 8).

Case three (specimen seven in Table 2): ERM with an area of 56.7 mm^2^, containing neovessels at stage V2. All neovessels and GCs were within the same layer. GFAP-positive cells were found around and beyond the leading capillaries (Figures 9). However, the direction of vessel growth and the orientation of GC processes did not coincide. The processes of GCs were unidirectional but did not form any clear structure.

Case four (specimen 11 in Table 2): ERM with an area of 97.8 mm^2^, containing a neovascular network at the advanced stage of angiogenesis (V3) with neovessels of very large caliber. Vitreoschisis was observed in the growth zone of the ERM periphery (Figure 10). A fragment of one vitreoschisis layer, removed and examined separately from the main specimen, contained GCs but no neovessels. Distal capillary loops were identified in the other ERM layer. Notably, GFAP-positive cells extended beyond the leading capillaries.

**FIGURE 11:**
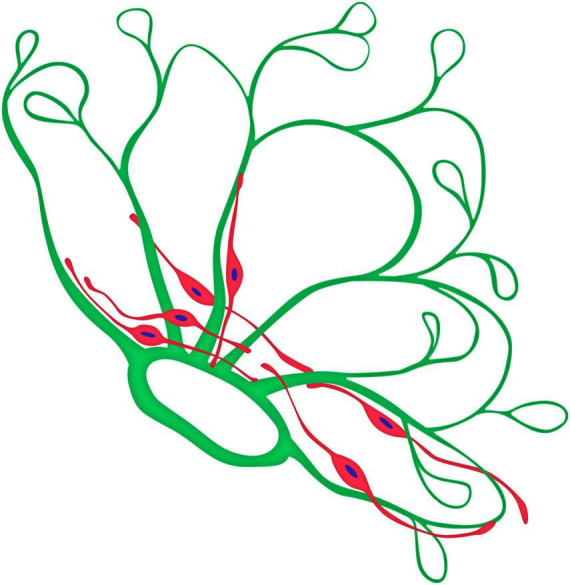
Graphical model of the topographical relationship between neovessels (green) and glial cells (red) of a neovascular cluster in PDR.

In summary, this study of whole-mounted ERMs with active neovessels identified GFAP-positive cells in 11 of 17 specimens. GCs had a distinct phenotype and characteristic distribution. Their specific topography, structure, and GFAP immunopositivity distinguished them from GFAP-negative hyalocytes. Additionally, GFAP-positive cells exhibited immunopositivity for type IV collagen. In all cases, most GCs were located at the base of neovascular clusters (Figure 11), where collagen density was greatest. In contrast, most ERMs lacked GCs in the growth zone (13 of 17 specimens). Among the 4 cases where GFAP-positive cells were detected in the growth zone, GCs were in contact with single loops of leading capillaries in two specimens. In one case, GCs were present in the vitreoschisis layer that contained no neovessels. Only in one specimen the growth zone capillaries and GCs were located within the same ERM layer. In cases where GCs were found in the growth zone (4 of 17 cases, 23%) and where glial cells extended beyond the vascularized areas (two of 17 cases, 12%), no distinct features indicated direct interaction between the leading capillaries and the GCs. The processes of GCs often were unidirectional but did not form any clear structure.

## 4 Discussion

It has been previously shown that fibrovascular membranes in patients with PDR may contain a significant number of GFAP-positive cells (Jerdan et al., 1986; Oberstein et al., 2011). Several authors have suggested that GC proliferation plays a major role in ERM formation in retinal diseases, including PDR (Bringmann and Wiedemann, 2009; Romaniuk et al., 2013). However, GCs represent only one of the many cellular components of ERMs (Heidenkummer and Kampik, 1992; Hiscott et al., 1984), and their numbers are often relatively low compared to the total cell population (Guidry, 2005). Moreover, ultrastructural and immunohistochemical studies have demonstrated that GCs are not consistently found in all surgical specimens of ERMs (Jerdan et al., 1986; Kim et al., 2015; Ohira and de Juan, 1990; Sramek et al., 1989). Notably, some studies reported that GFAP-immunoreactive cells were either not clearly detected in any of the ERMs containing neovessels (Hosoda et al., 1992; Snead et al., 2008) or their percentage relative to other cells types was minimal (= 5%) (Kwak et al., 1991). Hosoda et al. (1992) showed that neovessel formation in PDR was associated with collagen production, but no GCs were detected (Hosoda et al., 1992). Similarly, Kwak et al. (1991) reported that fibroblasts accounted for 93% of ERM cells in PDR (Kwak et al., 1991). The fibrous component of (collagen types I, III, and IV) was present in all studied cases, in contrast to glial component. Based on these findings, some authors suggested that GCs are not an essential cellular component in PDR (Kwak et al., 1991) and that “glial cells do not appear to play an important role during intravitreal neovascular tissue formation” (Hosoda et al., 1992). Therefore, there is yet no consensus on the role of GCs in the ERM in PDR. Interestingly, other authors have also shown that types I–VI collagen is an important component of membranes in PDR (Hamilton et al., 1982) and that fibroblasts are involved in the formation of both idiopathic ERMs and those associated with PDR. Fibroblasts are also present in the vitreous cortex of normal eyes (Sebag, 1992). However, the origin of fibroblasts in PDR remains undetermined (Boneva et al., 2021; Guidry, 1996; Kim et al., 2015).

Clinical and pathomorphological studies have shown that, unlike other common adult retinal diseases associated with ERM formation, the defining event in PDR is pathological angiogenesis. ERMs in PDR differ significantly from idiopathic ERMs in both cellular and molecular composition (Boneva et al., 2021). Apart from the *TGFB2* gene, no other genes have been identified whose expression in PDR changes in a manner similar to that observed in idiopathic ERMs. The pathogenesis of these diseases is most likely different, although both processes may have similar gene expression pathways (Tsotridou et al., 2020). It is also important to consider that not all ERMs excised during vitrectomy for PDR contain developing (or active) neovessels (Abu El-Asrar et al., 2010). These vessels can regress, for example, following laser photocoagulation. Remarkably, some studies on ERM structure in PDR do not mention neovessels at all, while others focus primarily on neovessel formation (Hamilton et al., 1982; Miller et al., 1984). Relatively few reports have examined the interaction between developing neovessels and GCs in PDR (Hosoda et al., 1992; Kim et al., 2015; Ohira and de Juan, 1990). Some authors suggested that GCs and their extracellular matrix may serve as a basis for neovessel growth (Nork et al., 1987; Ohira and de Juan, 1990). However, other findings indicate that collagen and fibronectin/laminin form a basement membrane-like structure that supports neovascular tissue formation (Hosoda et al., 1992; Jerdan et al., 1986). Thus, there is still no clear consensus on the role of glia in vascular development in PDR, opposite to what was shown in embryonic retinal angiogenesis (Chan-Ling et al., 2004; Dorrell et al., 2002; Fruttiger, 2002; Provis, 2001; Stone and Dreher, 1987).

The diversity of perspectives on ERM structure in PDR is reflected in the terminology used in the literature. Some authors classify epiretinal tissue as “gliosis” (Berufsverband der Augenärzte Deutschlands e.V. (BVA), Deutsche Ophthalmologische Gesellschaft (DOG), and Retinologische Gesellschaft e.V. (RG), 2021) or a “glial scar” (Bringmann and Wiedemann, 2009), while others refer to it as a “glio-vascular collagenous membrane” (Ohira and de Juan, 1990). Even in the early stages of PDR, when the ERM is transparent, it contains numerous fibroblasts. That is why Davis MD proposed to consider this process as fibrovascular. Interestingly, a similar debate exists in discussions of brain and spinal cord injuries, where scarring is commonly called “glial.” However, in these cases, the non-glial (connective tissue) component — fibroblasts and collagen — is also clearly expressed (Camand et al., 2004; Shearer and Fawcett, 2001).

In this study, we analyzed the topographic distribution of GCs within ERMs containing active neovessels in PDR, as well as their relationship to leading neovessels. During vitrectomy, entire neovessel clusters and the surrounding posterior hyaloid membrane were excised en bloc. We examined whole-mounted ERM preparations that included the zone of leading neovessels (“growth zone”). GFAP-positive cells were detected in 63% of ERM specimens. The following key features of neovessel and GC topography in ERMs in PDR were identified: (1). Leading capillaries were found at the periphery of neovascular networks, consistent with findings from clinical studies (Ishibazawa et al., 2016). (2). In all cases GCs tended to form clamps and were unevenly distributed in the ERM. All of them had a similar phenotype – bipolar shape, small body, and very long bidirectional cell processes; the topography and shape of GCs distinguished them from GFAP–negative hyalocytes. (3). The highest density of GFAP-positive cells was consistently observed near the epicenter of neovascular clusters. (4). In most ERMs, GFAP-positive cells were located in different focal planes from the neovessels, which has also been reported in previous studies (Hamilton et al., 1982; Ohira and de Juan, 1990). (5). In the majority of ERMs (13 of 17 specimens), glial cells were absent from the growth zone. In the remaining four cases (23%), GFAP-positive cells were observed around leading capillaries; however, no signs of direct interaction between terminal capillary loops and GCs were identified. In two of these four specimens (12%), the glial cells extended beyond the vascularized areas of the ERM, similarly to the results obtained by Ohira and de Juan (1990). (6). In one case with an advanced stage of angiogenesis (V3), there was vitreoschisis in the growth zone; the GFAP-positive cells were present only in one layer of the vitreoschisis which did not contain neovessels.

Thus, we found no evidence of direct interaction between glial cells and leading neovessels. The GFAP-positive glial cells we identified did not have the stellate shape characteristic of astrocytes; instead, their structure more closely resembled Müller cells. Several previous studies have also demonstrated the presence of Müller cells in ERMs associated with both PDR and idiopathic epiretinal membranes (Kase et al., 2006; Nork et al., 1987).

Most previous studies have analyzed ERMs using cross-sections. However, the selection of ERM fragments for cross-sectional analysis can significantly influence the results. Additionally, cross-sectional imaging makes it difficult to assess the spatial organization of cellular clusters or to identify distal segments of leading capillaries. In contrast, whole-mounted ERM preparations provide a more comprehensive view, overcoming these limitations. Previous studies using flat-mounted specimens have yielded valuable insights into ERM structure in normal eyes (Snead et al., 2002), macular holes (Gandorfer et al., 2009; Hisatomi et al., 2006; Schumann et al., 2011), idiopathic epiretinal fibrosis (Kohno et al., 2009; Snead et al., 2002), proliferative vitreoretinopathy, and PDR with active neovascularization (Oberstein et al., 2011). However, in the latter case, information on neovessels was not available. To our knowledge, the present study is the first to investigate whole-mounted ERM preparations in active PDR, including entire neovascular clusters. Furthermore, while previous studies have primarily relied on confocal microscopy, our findings demonstrate that ERMs with developing neovessels are thin and transparent enough to allow high-quality imaging with conventional fluorescence microscopy. Some discrepancies in ERM descriptions may be attributable to differences in the distance of analyzed ERM fragments from the neovascularization epicenter or the phase of PDR (i.e., whether neovessels are developing or regressing).

It has also been suggested that immunohistochemical studies may not always clearly distinguish between glial cells and hyalocytes (Schumann et al., 2011). Hyalocytes are known to express the monocyte/macrophage marker CD45, but not CD68 or GFAP (Sakamoto and Ishibashi, 2011; Schumann et al., 2011). In a study by Schumann et al. (2011) it was found that GFAP and CD45 were co-expressed in the same cells. The authors suggested that some GFAP-positive cells may actually be hyalocytes rather than GCs. Nonetheless, hyalocytes are not the only CD45-positive cells found in PDR. Some studies proposed that circulating CD45-positive fibrocytes contribute to ERM formation in PDR (Abu El-Asrar et al., 2015; Tamaki et al., 2016). Thus, the co-expression of GFAP and CD45 requires further investigation. It is known that during cellular transformation within ERMs, transitional cell forms may emerge (Vinores et al., 1990), emphasizing the need for further study of the morphological transformation of cells (Schumann et al., 2011).

Although we found no evidence of glial and neovascular interaction in the leading capillaries area, we assume that vascular evolution and GC proliferation are not completely independent processes. This conclusion is based on clinical observations and clinical-morphological comparisons in the studied cases. Pathological angiogenesis is the defining event in PDR, and extensive clinical data indicate that even during the natural course of PDR, the activity of the proliferative process is limited to a certain time period, averaging about 5 years (Beetham, 1963; Sdobnikova, 2021). Neovascular regression in PDR is commonly accompanied by progressively increasing fibrous proliferation. Ophthalmoscopic studies have shown that in the early stages of their evolution neovessels appear “bare,” but over time, whitish tissue becomes visible within the neovascular network (Danis and Davis, 2008; Davis, 1965). As the neovascular network matures or regresses, the fibrous component becomes more pronounced, manifesting ophthalmoscopically as “whitish fibers.” It is at this stage that tractional retinal detachment often occurs. The stages of neovascular regression are particularly evident following laser photocoagulation. Naturally, the development and regression of neovessels are diametrically opposite processes and are accompanied by different cellular events.

In our opinion, the presence of GCs around the epicenter of neovascularization and in the “growth zone” may reflect different phases of the process in PDR. In the first case, when GFAP-positive cells were localized at the base of the neovascular cluster, they could be directly involved in the involution of newly formed capillaries during vascular remodeling or regression, as these processes are typically accompanied by an increase in the fibrous component in PDR. The frequent presence of GCs in areas with higher collagen density and their immunoreactivity to type VI collagen, may indicate direct involvement of these cells in the fibrous transformation of epiretinal tissue. During neovessel regression, GFAP expression in GCs may decrease. It is plausible that persistent regression of neovessels may be accompanied by a reduction in GFAP expression, which then becomes undetectable. This assumption could explain the rare detection of GFAP-positive cells in PDR in published studies. It has been previously shown that Müller glial cells can acquire fibroblast-like properties, and it’s accompanied by a gradual decrease in GFAP expression (Guidry et al., 2009; Guidry, 2005; Luo et al., 2016; Vinores et al., 1990). In cases where the GCs were visualized in the “growth zone” (23%) or outside of it (12%), there was no evidence of their direct interaction with the leading capillaries. In these instances, GCs may serve functions unrelated to neovessel development, though the two processes could share common triggers. However, our assumption that GCs participate in fibrous transformation of ERMs is based only on indirect observations, which we acknowledge as a limitation of our study.

In our experience, when studying ERM in PDR, certain considerations may be useful: (1). Since ERM formation in PDR is closely associated with angiogenesis, it is important to determine the phase of vascular evolution (development or regression), which has distinct clinical features (Sdobnikova, 2021), as these phases are accompanied by different cellular events. (2). When analyzing ERM fragments, it is advisable to consider that in PDR their structure may differ depending on the distance from the epicenter of neovascularization in each cluster. (3). Greater insight can be gained from analyzing flat-mounted ERM specimens that include all zones of neovascular clusters. Moreover, when studying flat-mounted preparations, it is important to consider that ERM stratification is possible, as vitreoschisis layers in PDR may have different structure.

In the present we demonstrated that GCs have a recognizable phenotype and characteristic distribution. They also exhibited positive immunoreactivity for type IV collagen. Most of the GCs were located at the base of neovascular clusters, where the highest density of collagen fibrils was observed. GFAP-positive cells were identified around leading capillaries in 23% of cases, though no evidence of direct interaction between GCs and the leading capillaries was found. In cases where the GCs went beyond the vascularized areas, their processes were unidirectional but did not form any clear structure. The GFAP-positive glial cells identified in our study have not had the stellate shape characteristic of astrocytes and their structure was more consistent with morphology of Müller cells.

Since a fibrous component was present in all studied ERMs, whereas a glial component was not always detected, the terms “gliosis” and “glial scar” may not fully reflect the specificity of the proliferative process in PDR. In general, our results do not fundamentally contradict available reports regarding ERM structure in PDR, but rather indicate the need to standardize the approach to study PDR based on clinical-morphological correlation. The ocular structure and extraretinal neovascular growth in PDR provide this unique opportunity.

## 5 Conclusion

Our study found no direct interaction between neovessels and GCs around leading capillaries in PDR. However, in our opinion, vascular growth and GC proliferation are not independent events. The presence of GCs around the epicenter of neovascularization and within the “growth zone” may reflect different phases of proliferation in PDR. The presence of GFAP-positive cells at the base of neovascular clusters might indicate their direct involvement in the involution process of the newly developed capillaries during remodeling of the developing vascular network or during their regression. In cases where GCs were identified in the “growth zone” (23%) or outside of it (12%), we found no evidence indicating a direct interaction between the leading capillaries and the GCs. This suggests that GCs are involved in the processes that are not directly related to neovessel development, but pathological angiogenesis and proliferation of glial cells in PDR may have common triggers.

## Data Availability

The raw data supporting the conclusions of this article will be made available by the authors, without undue reservation.
